# Degradation of atorvastatin: (1*R*,2*S*,4*S*,5*S*)-4-(4-fluoro­phen­yl)-2-hydro­peroxy-4-hydr­oxy-2-isopropyl-*N*,5-diphenyl-3,6-dioxabicyclo­[3.1.0]hexane-1-carboxamide

**DOI:** 10.1107/S1600536808022265

**Published:** 2008-07-19

**Authors:** Muhammad Ashfaq, Muhammad Nawaz Tahir, Islam Ullah Khan, Mohammad S. Iqbal, Muhammad Nadeem Arshad

**Affiliations:** a Department of Chemistry, Government College University, Lahore, Pakistan; b Department of Physics, University of Sargodha, Sargodha, Pakistan

## Abstract

The degradation of atorvastatin calcium in methanol and hydrogen peroxide results in the crystallization of the title compound, C_26_H_24_FNO_6_, which shows several differences compared with the starting compound. In the crystal structure of the title compound, intra- and inter­molecular hydrogen bonding is found.

## Related literature

For related literature, see: Cremer & Pople (1975 ▶); Rouleau (2005 ▶); United States Pharmacopeia (2007 ▶).
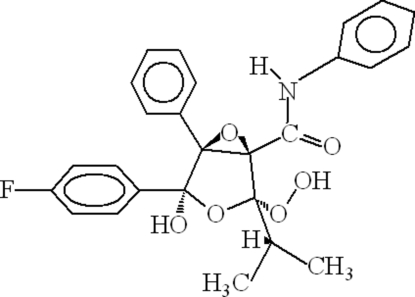


## Experimental

### 

#### Crystal data

C_26_H_24_FNO_6_
*M* *_r_* = 465.46
Monoclinic, 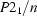

*a* = 11.7560 (6) Å
*b* = 11.7489 (6) Å
*c* = 17.0889 (9) Å
β = 94.438 (2)°
*V* = 2353.2 (2) Å^3^
*Z* = 4
Mo *K*α radiation radiation
μ = 0.10 mm^−1^
*T* = 296 (2) K
0.25 × 0.18 × 0.15 mm

#### Data collection

Bruker Kappa APEXII CCD diffractometer
Absorption correction: multi-scan (*SADABS*; Bruker, 2005 ▶) *T*_min_ = 0.975, *T*_max_ = 0.980
14754 measured reflections
5340 independent reflections
3008 reflections with *I* > 2σ(*I*)
*R*_int_ = 0.040

#### Refinement

*R*[*F* ^2^ > 2σ(*F* ^2^)] = 0.049
*wR*(*F* ^2^) = 0.136
*S* = 1.02
5340 reflections
318 parameters
H atoms treated by a mixture of independent and constrained refinement
Δρ_max_ = 0.34 e Å^−3^
Δρ_min_ = −0.28 e Å^−3^

### 

Data collection: *APEX2* (Bruker, 2007 ▶); cell refinement: *APEX2*; data reduction: *SAINT* (Bruker, 2007 ▶); program(s) used to solve structure: *SHELXS97* (Sheldrick, 2008 ▶); program(s) used to refine structure: *SHELXL97* (Sheldrick, 2008 ▶); molecular graphics: *ORTEP-3 for Windows* (Farrugia, 1997 ▶) and *PLATON* (Spek, 2003 ▶); software used to prepare material for publication: *WinGX* (Farrugia, 1999 ▶) and *PLATON*.

## Supplementary Material

Crystal structure: contains datablocks global, I. DOI: 10.1107/S1600536808022265/nc2108sup1.cif

Structure factors: contains datablocks I. DOI: 10.1107/S1600536808022265/nc2108Isup2.hkl

Additional supplementary materials:  crystallographic information; 3D view; checkCIF report

## Figures and Tables

**Table 1:** Hydrogen-bond geometry (Å, °)

*D*—H⋯*A*	*D*—H	H⋯*A*	*D*⋯*A*	*D*—H⋯*A*
N1—H1⋯O3	0.86 (2)	2.36 (2)	2.780 (2)	110.8 (17)
N1—H1⋯O2^i^	0.86 (2)	2.37 (2)	3.216 (2)	168.0 (18)
O2—H2⋯O5	0.84 (2)	2.15 (2)	2.920 (2)	152 (2)
O2—H2⋯O3^ii^	0.84 (2)	2.35 (2)	2.8188 (18)	116.1 (18)
O5—H5⋯O6	0.82	1.99	2.655 (2)	138

Symmetry codes: (i) 
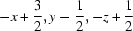
; (ii) 
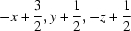
.

**Fig. 1.**
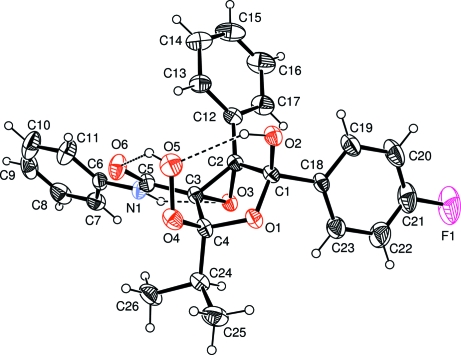
ORTEP drawing of the title compound, with the atom numbering scheme. The thermal ellipsoids are drawn at the 30% probability level. H-atoms are shown by small circles of arbitrary radii. The dashed lines shows intramolecular H-bonding.

**Fig. 2.**
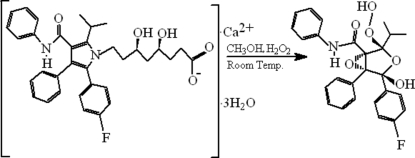
The formation of the title compound.

## supplementary crystallographic information

### Comment

Atorvastatin calcium is widly used as synthetic lipid-lowering agent (Rouleau, 2005). The medicinal organic compounds are affected by the environment in which they are stored and produce degradation products through reactions with moisture (humidity) and oxygen in air (oxidation) or due to thermal shocks. Thus, all the drug substances are given an expiration date which is the time at which 10% of the initial amount of a drug is transformed to various degradation products (United States Pharmacopeia, 2007). Thus it is a standard practice to study the stability profile of a drug substance under stress. In order to simulate the air oxidation under accelerated conditions the products are subjected to the reaction with hydrogen peroxide. The purpose of this study was to see the reaction of atorvastatin calcium towards hydrogen peroxide. In this example, the title compound crystallized after the reaction at ambient temperature.

In the structure of the title compound, the central five-membered ring (O1/C1–C4) is in an envelope conformation, with the C1–C4 atoms in the plane (Fig. 1). The puckering parameters (Cremer & Pople, 1975) are Q = 0.9737 (16)Å, θ = 115.69 (10)° and φ = 0.10 (13)°. The dihedral angles between this ring and benzene rings C6–C11, C12–C17 and C18–C23 are 88.71 (11), 66.85 (11) and 64.39 (12)°, respectively. There is intramolecular O—H···O and N—H···O hydrogen bonding between N1 and O3, between O2 and O5 and between O5 and O6 (Fig. 1 and Table 1). In the crystal structure, the molecules are connected *via* intermolecular O—H···O and N—H···O hydrogen bonding (Table 1).

### Experimental

Atorvastatin calcium (100 mg) was dissolved in methanol (25 ml) at room temperature. A separate solution (10 ml) of hydrogen peroxide (5%) was prepared in distilled water. Both the solutions were mixed togather and set aside for 2 months. The crystals suitable for *x*-ray diffraction of the title compound (I) were obtained by filteration.

### Refinement

The coordinates of H atoms attached with N1 and O2 were refined freely. The remaining H atoms were positioned with idealized geometry (O-H allowed to rotate but not to tip) with C—H = 0.93, 0.96 Å and O—H = 0.82 Å for aromatic, methyl and peroxide H, and were refined using a riding model with *U*_iso_(H) = *xU*_eq_(C, N, O), where *x* = 1.5 for methyl H, and *x* = 1.2 for all other H atoms.

### Crystal data

**Table:**

C_26_H_24_FNO_6_	*F*_000_ = 976
*M**_r_* = 465.46	*D*_x_ = 1.314 Mg m^−^^3^
Monoclinic, *P*2_1_/*n*	Mo *K*α radiation radiation λ = 0.71073 Å
Hall symbol: -P 2yn	Cell parameters from 5340 reflections
*a* = 11.7560 (6) Å	θ = 2.0–27.5º
*b* = 11.7489 (6) Å	µ = 0.10 mm^−^^1^
*c* = 17.0889 (9) Å	*T* = 296 (2) K
β = 94.438 (2)º	Prismatic, colorless
*V* = 2353.2 (2) Å^3^	0.25 × 0.18 × 0.15 mm
*Z* = 4	

### Data collection

**Table:**

Bruker Kappa APEXII CCD diffractometer	5340 independent reflections
Radiation source: fine-focus sealed tube	3008 reflections with *I* > 2σ(*I*)
Monochromator: graphite	*R*_int_ = 0.040
Detector resolution: 7.50 pixels mm^-1^	θ_max_ = 27.5º
*T* = 296(2) K	θ_min_ = 2.0º
ω scans	*h* = −15→14
Absorption correction: multi-scan(SADABS; Bruker, 2005)	*k* = −15→8
*T*_min_ = 0.975, *T*_max_ = 0.980	*l* = −22→21
14754 measured reflections	

### Refinement

**Table:**

Refinement on *F*^2^	Hydrogen site location: inferred from neighbouring sites
Least-squares matrix: full	H atoms treated by a mixture of independent and constrained refinement
*R*[*F*^2^ > 2σ(*F*^2^)] = 0.049	*w* = 1/[σ^2^(*F*_o_^2^) + (0.0541*P*)^2^ + 0.5969*P*] where *P* = (*F*_o_^2^ + 2*F*_c_^2^)/3
*wR*(*F*^2^) = 0.136	(Δ/σ)_max_ < 0.001
*S* = 1.02	Δρ_max_ = 0.34 e Å^−^^3^
5340 reflections	Δρ_min_ = −0.27 e Å^−^^3^
318 parameters	Extinction correction: emperical, Fc^*^=kFc[1+0.001xFc^2^λ^3^/sin(2θ)]^-1/4^
Primary atom site location: structure-invariant direct methods	Extinction coefficient: 0032 (7)
Secondary atom site location: difference Fourier map	

### Special details

**Table:**

Geometry. Bond distances, angles *etc*. have been calculated using the rounded fractional coordinates. All su's are estimated from the variances of the (full) variance-covariance matrix. The cell e.s.d.'s are taken into account in the estimation of distances, angles and torsion angles
Refinement. Refinement of *F*^2^ against ALL reflections. The weighted *R*-factor *wR* and goodness of fit *S* are based on *F*^2^, conventional *R*-factors *R* are based on *F*, with *F* set to zero for negative *F*^2^. The threshold expression of *F*^2^ > σ(*F*^2^) is used only for calculating *R*-factors(gt) *etc*. and is not relevant to the choice of reflections for refinement. *R*-factors based on *F*^2^ are statistically about twice as large as those based on *F*, and *R*-factors based on ALL data will be even larger.

### Fractional atomic coordinates and isotropic or equivalent isotropic displacement parameters (Å^2^)

**Table:**

	*x*	*y*	*z*	*U*_iso_*/*U*_eq_	
F1	0.7275 (2)	0.00876 (18)	−0.10244 (10)	0.1428 (12)	
O1	0.91061 (11)	0.21012 (11)	0.21795 (7)	0.0426 (5)	
O2	0.78009 (12)	0.35021 (11)	0.17484 (8)	0.0455 (5)	
O3	0.75203 (11)	0.07280 (10)	0.26940 (7)	0.0383 (4)	
O4	0.96555 (12)	0.28576 (11)	0.34067 (8)	0.0512 (5)	
O5	0.88438 (14)	0.38004 (12)	0.33419 (9)	0.0591 (6)	
O6	0.78216 (14)	0.25802 (13)	0.43917 (8)	0.0622 (6)	
N1	0.70884 (15)	0.07964 (14)	0.42697 (10)	0.0428 (6)	
C1	0.79736 (16)	0.23366 (15)	0.18667 (11)	0.0373 (6)	
C2	0.72161 (16)	0.18934 (14)	0.24910 (11)	0.0361 (6)	
C3	0.79830 (16)	0.16319 (15)	0.31885 (11)	0.0362 (6)	
C4	0.91978 (17)	0.19004 (16)	0.30000 (11)	0.0398 (6)	
C5	0.76193 (17)	0.17068 (16)	0.40107 (11)	0.0426 (7)	
C6	0.66547 (17)	0.06482 (18)	0.50124 (11)	0.0430 (7)	
C7	0.64405 (19)	−0.0442 (2)	0.52493 (13)	0.0545 (8)	
C8	0.5989 (2)	−0.0630 (3)	0.59596 (16)	0.0742 (10)	
C9	0.5747 (2)	0.0265 (3)	0.64262 (15)	0.0848 (13)	
C10	0.5957 (3)	0.1338 (3)	0.61935 (17)	0.0905 (14)	
C11	0.6417 (2)	0.1550 (2)	0.54889 (15)	0.0731 (10)	
C12	0.60000 (17)	0.22332 (16)	0.24931 (12)	0.0432 (7)	
C13	0.56860 (19)	0.31146 (18)	0.29682 (14)	0.0535 (8)	
C14	0.4566 (2)	0.3464 (2)	0.29578 (17)	0.0691 (10)	
C15	0.3765 (2)	0.2949 (2)	0.2464 (2)	0.0809 (13)	
C16	0.4051 (2)	0.2077 (3)	0.1996 (2)	0.0851 (13)	
C17	0.5172 (2)	0.1703 (2)	0.20123 (15)	0.0634 (9)	
C18	0.77882 (17)	0.17385 (16)	0.10816 (11)	0.0417 (7)	
C19	0.6918 (2)	0.2070 (2)	0.05454 (13)	0.0628 (9)	
C20	0.6729 (3)	0.1504 (3)	−0.01611 (15)	0.0865 (13)	
C21	0.7429 (3)	0.0629 (3)	−0.03176 (16)	0.0877 (13)	
C22	0.8274 (3)	0.0266 (2)	0.01933 (17)	0.0858 (13)	
C23	0.8452 (2)	0.08283 (19)	0.09060 (14)	0.0633 (9)	
C24	1.00575 (18)	0.09376 (19)	0.31729 (13)	0.0483 (8)	
C25	1.1220 (2)	0.1214 (2)	0.28961 (16)	0.0707 (10)	
C26	1.0152 (2)	0.0571 (2)	0.40307 (14)	0.0725 (10)	
H1	0.7025 (18)	0.0224 (18)	0.3959 (12)	0.0513*	
H2	0.7978 (19)	0.3806 (18)	0.2186 (13)	0.0546*	
H5	0.84238	0.37658	0.37026	0.0709*	
H7	0.65998	−0.10545	0.49308	0.0653*	
H8	0.58498	−0.13698	0.61205	0.0891*	
H9	0.54382	0.01370	0.69032	0.1016*	
H10	0.57890	0.19449	0.65141	0.1082*	
H11	0.65640	0.22924	0.53375	0.0877*	
H13	0.62383	0.34762	0.32991	0.0642*	
H14	0.43608	0.40471	0.32866	0.0830*	
H15	0.30121	0.31969	0.24457	0.0971*	
H16	0.34924	0.17291	0.16623	0.1022*	
H17	0.53617	0.10956	0.16989	0.0761*	
H19	0.64536	0.26790	0.06595	0.0754*	
H20	0.61343	0.17187	−0.05218	0.1038*	
H22	0.87298	−0.03469	0.00730	0.1031*	
H23	0.90295	0.05841	0.12698	0.0760*	
H24	0.9756 (19)	0.0315 (18)	0.2877 (12)	0.0580*	
H25A	1.17269	0.05848	0.30093	0.1061*	
H25B	1.15192	0.18810	0.31628	0.1061*	
H25C	1.11487	0.13507	0.23406	0.1061*	
H26A	1.06946	−0.00373	0.41036	0.1087*	
H26B	0.94205	0.03156	0.41737	0.1087*	
H26C	1.03999	0.12047	0.43554	0.1087*	

### Atomic displacement parameters (Å^2^)

**Table:**

	*U*^11^	*U*^22^	*U*^33^	*U*^12^	*U*^13^	*U*^23^
F1	0.231 (3)	0.1278 (16)	0.0649 (12)	−0.0243 (16)	−0.0177 (13)	−0.0448 (11)
O1	0.0387 (8)	0.0528 (8)	0.0357 (8)	−0.0054 (6)	−0.0016 (6)	0.0093 (6)
O2	0.0619 (10)	0.0324 (7)	0.0409 (8)	−0.0028 (6)	−0.0034 (7)	0.0037 (6)
O3	0.0471 (8)	0.0307 (6)	0.0361 (7)	−0.0042 (6)	−0.0036 (6)	0.0030 (5)
O4	0.0506 (9)	0.0456 (8)	0.0549 (9)	−0.0083 (7)	−0.0120 (7)	−0.0004 (7)
O5	0.0700 (11)	0.0434 (8)	0.0632 (11)	−0.0068 (8)	0.0008 (8)	−0.0020 (7)
O6	0.0906 (12)	0.0504 (9)	0.0464 (9)	−0.0176 (8)	0.0105 (8)	−0.0098 (7)
N1	0.0522 (11)	0.0422 (9)	0.0347 (10)	−0.0061 (8)	0.0080 (8)	−0.0016 (7)
C1	0.0396 (12)	0.0348 (10)	0.0362 (11)	−0.0016 (8)	−0.0044 (8)	0.0063 (8)
C2	0.0416 (12)	0.0293 (9)	0.0364 (11)	−0.0029 (8)	−0.0024 (8)	0.0022 (8)
C3	0.0414 (11)	0.0322 (9)	0.0339 (11)	−0.0048 (8)	−0.0032 (8)	0.0015 (8)
C4	0.0437 (12)	0.0412 (10)	0.0329 (11)	−0.0068 (9)	−0.0064 (9)	0.0042 (8)
C5	0.0492 (12)	0.0423 (11)	0.0357 (11)	−0.0021 (9)	−0.0004 (9)	0.0002 (9)
C6	0.0379 (12)	0.0577 (12)	0.0334 (11)	−0.0021 (10)	0.0024 (9)	0.0001 (10)
C7	0.0486 (13)	0.0631 (14)	0.0525 (14)	0.0022 (11)	0.0090 (11)	0.0138 (11)
C8	0.0561 (16)	0.103 (2)	0.0640 (17)	−0.0041 (14)	0.0080 (13)	0.0336 (16)
C9	0.0597 (18)	0.156 (3)	0.0398 (15)	−0.0171 (19)	0.0110 (12)	0.0042 (18)
C10	0.087 (2)	0.124 (3)	0.0645 (19)	−0.0228 (19)	0.0319 (16)	−0.0354 (18)
C11	0.0829 (19)	0.0770 (17)	0.0628 (17)	−0.0140 (14)	0.0274 (14)	−0.0194 (14)
C12	0.0416 (12)	0.0378 (10)	0.0497 (12)	−0.0045 (9)	−0.0002 (10)	0.0081 (9)
C13	0.0451 (14)	0.0472 (12)	0.0682 (16)	0.0003 (10)	0.0045 (11)	0.0015 (11)
C14	0.0549 (16)	0.0530 (14)	0.101 (2)	0.0052 (12)	0.0171 (15)	0.0081 (13)
C15	0.0405 (15)	0.0652 (17)	0.137 (3)	0.0015 (13)	0.0072 (16)	0.0209 (18)
C16	0.0458 (16)	0.0774 (19)	0.128 (3)	−0.0130 (14)	−0.0190 (16)	−0.0045 (18)
C17	0.0509 (15)	0.0561 (14)	0.0810 (18)	−0.0072 (12)	−0.0098 (12)	−0.0047 (12)
C18	0.0531 (13)	0.0367 (10)	0.0343 (11)	−0.0045 (9)	−0.0031 (9)	0.0058 (8)
C19	0.0784 (18)	0.0555 (14)	0.0507 (14)	0.0011 (12)	−0.0196 (12)	0.0020 (11)
C20	0.120 (3)	0.080 (2)	0.0526 (17)	−0.0065 (18)	−0.0373 (16)	−0.0018 (14)
C21	0.142 (3)	0.0704 (19)	0.0476 (16)	−0.0215 (19)	−0.0116 (18)	−0.0167 (14)
C22	0.126 (3)	0.0653 (17)	0.0657 (19)	0.0120 (17)	0.0044 (18)	−0.0192 (14)
C23	0.0869 (19)	0.0527 (13)	0.0489 (14)	0.0085 (13)	−0.0044 (12)	−0.0052 (11)
C24	0.0447 (13)	0.0498 (12)	0.0488 (14)	0.0012 (10)	−0.0073 (10)	0.0072 (10)
C25	0.0432 (14)	0.0843 (18)	0.0840 (19)	0.0059 (12)	0.0013 (13)	0.0167 (14)
C26	0.0714 (17)	0.0825 (18)	0.0613 (16)	0.0128 (14)	−0.0092 (13)	0.0265 (13)

### Geometric parameters (Å, °)

**Table:**

F1—C21	1.365 (3)	C15—C16	1.358 (4)
O1—C1	1.423 (2)	C16—C17	1.387 (3)
O1—C4	1.418 (2)	C18—C19	1.376 (3)
O2—C1	1.397 (2)	C18—C23	1.371 (3)
O3—C2	1.450 (2)	C19—C20	1.381 (4)
O3—C3	1.438 (2)	C20—C21	1.356 (5)
O4—O5	1.461 (2)	C21—C22	1.340 (5)
O4—C4	1.407 (2)	C22—C23	1.387 (4)
O6—C5	1.229 (2)	C24—C25	1.516 (3)
O2—H2	0.84 (2)	C24—C26	1.524 (3)
O5—H5	0.8200	C7—H7	0.9300
N1—C5	1.331 (3)	C8—H8	0.9300
N1—C6	1.415 (3)	C9—H9	0.9300
N1—H1	0.86 (2)	C10—H10	0.9300
C1—C18	1.515 (3)	C11—H11	0.9300
C1—C2	1.533 (3)	C13—H13	0.9300
C2—C3	1.470 (3)	C14—H14	0.9300
C2—C12	1.485 (3)	C15—H15	0.9300
C3—C4	1.521 (3)	C16—H16	0.9300
C3—C5	1.503 (3)	C17—H17	0.9300
C4—C24	1.530 (3)	C19—H19	0.9300
C6—C11	1.378 (3)	C20—H20	0.9300
C6—C7	1.373 (3)	C22—H22	0.9300
C7—C8	1.380 (3)	C23—H23	0.9300
C8—C9	1.363 (5)	C24—H24	0.94 (2)
C9—C10	1.350 (5)	C25—H25A	0.9600
C10—C11	1.380 (4)	C25—H25B	0.9600
C12—C17	1.374 (3)	C25—H25C	0.9600
C12—C13	1.384 (3)	C26—H26A	0.9600
C13—C14	1.378 (3)	C26—H26B	0.9600
C14—C15	1.357 (4)	C26—H26C	0.9600
			
C1—O1—C4	113.66 (14)	C18—C19—C20	120.5 (2)
C2—O3—C3	61.20 (11)	C19—C20—C21	118.6 (3)
O5—O4—C4	110.25 (14)	F1—C21—C20	119.1 (3)
C1—O2—H2	105.3 (14)	F1—C21—C22	118.0 (3)
O4—O5—H5	109.00	C20—C21—C22	122.9 (3)
C5—N1—C6	127.44 (17)	C21—C22—C23	118.3 (3)
C5—N1—H1	116.4 (14)	C18—C23—C22	120.9 (2)
C6—N1—H1	116.1 (14)	C4—C24—C26	113.12 (18)
O1—C1—C2	104.45 (14)	C25—C24—C26	111.13 (19)
O1—C1—C18	107.99 (15)	C4—C24—C25	112.29 (18)
O1—C1—O2	111.48 (15)	C6—C7—H7	120.00
O2—C1—C2	110.37 (15)	C8—C7—H7	120.00
O2—C1—C18	108.49 (15)	C7—C8—H8	120.00
C2—C1—C18	114.03 (15)	C9—C8—H8	120.00
O3—C2—C3	58.97 (11)	C8—C9—H9	120.00
O3—C2—C12	118.22 (15)	C10—C9—H9	120.00
C1—C2—C3	106.41 (15)	C9—C10—H10	119.00
O3—C2—C1	109.96 (14)	C11—C10—H10	119.00
C1—C2—C12	121.45 (16)	C6—C11—H11	120.00
C3—C2—C12	125.76 (17)	C10—C11—H11	120.00
O3—C3—C5	118.09 (15)	C12—C13—H13	120.00
C4—C3—C5	121.71 (16)	C14—C13—H13	120.00
C2—C3—C4	108.17 (15)	C13—C14—H14	120.00
C2—C3—C5	123.01 (16)	C15—C14—H14	120.00
O3—C3—C2	59.83 (11)	C14—C15—H15	120.00
O3—C3—C4	110.33 (15)	C16—C15—H15	120.00
O4—C4—C24	105.89 (16)	C15—C16—H16	120.00
C3—C4—C24	115.00 (16)	C17—C16—H16	120.00
O1—C4—C24	108.21 (16)	C12—C17—H17	120.00
O1—C4—O4	110.64 (15)	C16—C17—H17	120.00
O1—C4—C3	104.07 (15)	C18—C19—H19	120.00
O4—C4—C3	112.99 (15)	C20—C19—H19	120.00
O6—C5—N1	124.85 (18)	C19—C20—H20	121.00
N1—C5—C3	116.01 (16)	C21—C20—H20	121.00
O6—C5—C3	119.15 (17)	C21—C22—H22	121.00
N1—C6—C11	122.63 (19)	C23—C22—H22	121.00
C7—C6—C11	119.54 (19)	C18—C23—H23	120.00
N1—C6—C7	117.81 (18)	C22—C23—H23	120.00
C6—C7—C8	120.1 (2)	C4—C24—H24	104.8 (14)
C7—C8—C9	120.2 (3)	C25—C24—H24	108.0 (14)
C8—C9—C10	119.8 (3)	C26—C24—H24	107.0 (13)
C9—C10—C11	121.2 (3)	C24—C25—H25A	109.00
C6—C11—C10	119.2 (2)	C24—C25—H25B	109.00
C2—C12—C13	120.23 (18)	C24—C25—H25C	109.00
C2—C12—C17	120.97 (18)	H25A—C25—H25B	110.00
C13—C12—C17	118.8 (2)	H25A—C25—H25C	109.00
C12—C13—C14	120.9 (2)	H25B—C25—H25C	109.00
C13—C14—C15	119.4 (2)	C24—C26—H26A	109.00
C14—C15—C16	120.7 (2)	C24—C26—H26B	109.00
C15—C16—C17	120.4 (3)	C24—C26—H26C	109.00
C12—C17—C16	119.8 (2)	H26A—C26—H26B	109.00
C19—C18—C23	118.79 (19)	H26A—C26—H26C	109.00
C1—C18—C19	120.47 (18)	H26B—C26—H26C	109.00
C1—C18—C23	120.69 (18)		
			
C4—O1—C1—O2	100.71 (17)	O3—C3—C4—O4	173.71 (14)
C4—O1—C1—C2	−18.48 (18)	O3—C3—C4—C24	−64.6 (2)
C4—O1—C1—C18	−140.20 (15)	C2—C3—C4—O1	−10.10 (19)
C1—O1—C4—O4	−103.48 (17)	C2—C3—C4—O4	109.97 (17)
C1—O1—C4—C3	18.17 (19)	C2—C3—C4—C24	−128.29 (17)
C1—O1—C4—C24	140.93 (16)	C5—C3—C4—O1	−161.36 (16)
C3—O3—C2—C1	97.59 (16)	C5—C3—C4—O4	−41.3 (2)
C3—O3—C2—C12	−116.82 (19)	C5—C3—C4—C24	80.5 (2)
C2—O3—C3—C4	−99.75 (16)	O3—C3—C5—O6	−166.15 (17)
C2—O3—C3—C5	113.84 (19)	O3—C3—C5—N1	13.7 (3)
O5—O4—C4—O1	68.46 (18)	C2—C3—C5—O6	−95.6 (2)
O5—O4—C4—C3	−47.78 (19)	C2—C3—C5—N1	84.3 (2)
O5—O4—C4—C24	−174.52 (14)	C4—C3—C5—O6	51.4 (3)
C6—N1—C5—O6	0.3 (3)	C4—C3—C5—N1	−128.75 (19)
C6—N1—C5—C3	−179.54 (18)	O1—C4—C24—C25	58.3 (2)
C5—N1—C6—C7	−161.4 (2)	O1—C4—C24—C26	−174.90 (17)
C5—N1—C6—C11	20.3 (3)	O4—C4—C24—C25	−60.3 (2)
O1—C1—C2—O3	−51.65 (17)	O4—C4—C24—C26	66.5 (2)
O1—C1—C2—C3	10.65 (18)	C3—C4—C24—C25	174.17 (18)
O1—C1—C2—C12	164.07 (15)	C3—C4—C24—C26	−59.1 (2)
O2—C1—C2—O3	−171.59 (14)	N1—C6—C7—C8	−178.3 (2)
O2—C1—C2—C3	−109.29 (16)	C11—C6—C7—C8	0.1 (3)
O2—C1—C2—C12	44.1 (2)	N1—C6—C11—C10	177.7 (2)
C18—C1—C2—O3	66.0 (2)	C7—C6—C11—C10	−0.6 (4)
C18—C1—C2—C3	128.30 (16)	C6—C7—C8—C9	0.4 (4)
C18—C1—C2—C12	−78.3 (2)	C7—C8—C9—C10	−0.4 (4)
O1—C1—C18—C19	−160.88 (18)	C8—C9—C10—C11	−0.1 (4)
O1—C1—C18—C23	21.9 (2)	C9—C10—C11—C6	0.6 (4)
O2—C1—C18—C19	−39.9 (2)	C2—C12—C13—C14	177.9 (2)
O2—C1—C18—C23	142.86 (19)	C17—C12—C13—C14	−0.5 (3)
C2—C1—C18—C19	83.5 (2)	C2—C12—C17—C16	−176.6 (2)
C2—C1—C18—C23	−93.7 (2)	C13—C12—C17—C16	1.7 (4)
O3—C2—C3—C4	103.43 (15)	C12—C13—C14—C15	−1.2 (4)
O3—C2—C3—C5	−105.77 (18)	C13—C14—C15—C16	1.7 (4)
C1—C2—C3—O3	−103.78 (15)	C14—C15—C16—C17	−0.4 (5)
C1—C2—C3—C4	−0.35 (19)	C15—C16—C17—C12	−1.3 (4)
C1—C2—C3—C5	150.45 (16)	C1—C18—C19—C20	−178.2 (2)
C12—C2—C3—O3	104.29 (19)	C23—C18—C19—C20	−0.9 (3)
C12—C2—C3—C4	−152.28 (17)	C1—C18—C23—C22	179.0 (2)
C12—C2—C3—C5	−1.5 (3)	C19—C18—C23—C22	1.7 (3)
O3—C2—C12—C13	121.7 (2)	C18—C19—C20—C21	−1.0 (4)
O3—C2—C12—C17	−60.0 (3)	C19—C20—C21—F1	−177.8 (3)
C1—C2—C12—C13	−96.8 (2)	C19—C20—C21—C22	2.2 (5)
C1—C2—C12—C17	81.5 (2)	F1—C21—C22—C23	178.6 (3)
C3—C2—C12—C13	51.3 (3)	C20—C21—C22—C23	−1.4 (5)
C3—C2—C12—C17	−130.4 (2)	C21—C22—C23—C18	−0.6 (4)
O3—C3—C4—O1	53.64 (18)		

### Hydrogen-bond geometry (Å, °)

**Table:**

*D*—H···*A*	*D*—H	H···*A*	*D*···*A*	*D*—H···*A*
N1—H1···O3	0.86 (2)	2.36 (2)	2.780 (2)	110.8 (17)
N1—H1···O2^i^	0.86 (2)	2.37 (2)	3.216 (2)	168.0 (18)
O2—H2···O5	0.84 (2)	2.15 (2)	2.920 (2)	152 (2)
O2—H2···O3^ii^	0.84 (2)	2.35 (2)	2.8188 (18)	116.1 (18)
O5—H5···O6	0.8200	1.9900	2.655 (2)	138.00

Symmetry codes: (i) −*x*+3/2, *y*−1/2, −*z*+1/2; (ii) −*x*+3/2, *y*+1/2, −*z*+1/2.

## References

[bb1] Bruker (2005). *SADABS* Bruker AXS Inc., Madison, Wisconsin, USA.

[bb2] Bruker (2007). *APEX2* and *SAINT* Bruker AXS Inc., Madison, Wisconsin, USA.

[bb3] Cremer, D. & Pople, J. A. (1975). *J. Am. Chem. Soc.* **97**, 1354–1358.

[bb4] Farrugia, L. J. (1997). *J. Appl. Cryst.* **30**, 565.

[bb5] Farrugia, L. J. (1999). *J. Appl. Cryst.* **32**, 837–838.

[bb6] United States Pharmacopeia (2007). *United States Pharmacopoeia*, 2nd ed. Rockville: United States Pharmacopial Convention.

[bb7] Rouleau, J. (2005). *Am. J. Med.* **118** (Suppl. 12A), 28–35. 10.1016/j.amjmed.2005.09.014 16356805

[bb8] Sheldrick, G. M. (2008). *Acta Cryst.* A**64**, 112–122. 10.1107/S0108767307043930 18156677

[bb9] Spek, A. L. (2003). *J. Appl. Cryst.* **36**, 7–13.

